# Genetic Variants in the Mitochondrial Thymidylate Biosynthesis Pathway Increase Colorectal Cancer Risk

**DOI:** 10.3390/curroncol30090583

**Published:** 2023-08-30

**Authors:** Entesar M. Arrait, Ayat B. Al-Ghafari, Huda A. Al Doghaither

**Affiliations:** 1 Biochemistry Department, Faculty of Science, King Abdulaziz University, Jeddah 21589, Saudi Arabia; earrait0001@stu.kau.edu.sa (E.M.A.); haldoghaither@kau.edu.sa (H.A.A.D.); 2 Experimental Biochemistry Unit, King Fahd Medical Research Center, King Abdulaziz University, Jeddah 22252, Saudi Arabia; 3 Cancer and Mutagenesis Research Unit, King Fahd Medical Research Center, King Abdulaziz University, Jeddah 22252, Saudi Arabia

**Keywords:** colorectal cancer, capecitabine, CDA, TP, MTHFR, genetic variants

## Abstract

We assess the contributions of genetic variants for the enzymes involved in capecitabine metabolism to colorectal cancer (CRC) development risk. In this case-control study, DNA samples were collected from 66 patients (King Abdulaziz University Hospital) and 65 controls (King Fahad General Hospital) between April and November 2022 to be used in PCR-RFLP. The chi-square (χ^2^) test at a significance level of *p* ˂ 0.05 was used to estimate genotype and allele frequencies. The Lys27Gln variant of cytidine deaminase (CDA) showed a risk ratio (RR) of 1.47 for heterozygous (AC) carriers, with genotype distributions for patients (χ^2^ = 1.97) and controls (χ^2^ = 14.7). Homozygous (AA) Ala70Thr carriers demonstrated a three-fold higher risk, with genotype distributions for patients (χ^2^ = 3.85) and controls (χ^2^ = 4.23). Genotype distributions of the 5,10-methylenetetrahydrofolate reductase (MTHFR) C677T variant for patients were (χ^2^ = 22.43) and for controls were (χ^2^ = 0.07); for the *MTHFR* A1298C variant, they were (χ^2^ = 54.44) for patients and (χ^2^ = 4.58) for controls. Heterozygous (AC) carriers of the A1298C variant demonstrated highly significant protection against CRC development (RR = 0.2, *p* = 0.001), while a two-fold higher risk for CRC was estimated for homozygous genotype (CC) carriers. In conclusion, the heterozygous genotype of *CDA* Lys27Gln, the homozygous genotype of *CDA* Ala70Thr, and the homozygous genotype of *MTHFR* A1298C were associated with CRC development risk. The heterozygous genotype of *MTHFR* A1298C variant provided highly significant protection against CRC development. Further examinations using a larger population size are needed to reliably confirm our findings.

## 1. Introduction

Colorectal cancer (CRC) is a heterogeneous disease caused by the abnormal proliferation of epithelial cells, resulting in the formation of benign polyps in the intestinal mucosa of the colon or rectum. Untreated polyps may progressively develop into cancer [1]. Globally, according to GLOBOCAN 2020, CRC is the third most common type of cancer after breast and lung cancers and the second leading cause of cancer-related mortality among both genders [2]. In Saudi Arabia, CRC is considered the most common type of cancer in men and the third most common cancer type among women [3]. The development of CRC was related to multiple signaling pathways, including the cell cycle, immunity, aging, and metabolism [4]. Generally, the treatment of CRC depends on the stage of the tumor. Chemotherapy, among other available treatment options, remains the most used treatment for all CRC stages. Chemotherapeutic drugs are classified according to many factors such as chemical structure, their relationship to other drugs, and their mode of action. However, the most common classification is based on drugs mode of action such as topoisomerase inhibitors (e.g., irinotecan), alkylating agents (e.g., oxaliplatin), antitumor antibiotics (e.g., doxorubicin), mitotic inhibitors (e.g., vincristine), and antimetabolite (e.g., capecitabine) [5]. Capecitabine (Xeloda^®^) is one of the most used chemotherapeutic agents in the treatment of advanced metastatic CRC cases (stages III and IV). Capecitabine is a fluoropyrimidine carbamate with anticancer and antimetabolite properties that interferes with the synthesis of DNA and RNA and therefore inhibits the growth of tumor cells and the formation of proteins via its active metabolite, 5-fluorouracil (5-FU) [6]. In comparison to other chemotherapeutic agents, it causes fewer adverse side effects and enhances the relapse-free survival in CRC patients. Moreover, it results in acceptable tolerability, especially in elderly patients [6]. Capecitabine metabolism involves three major steps. Firstly, it is converted to intermediate 5′-deoxy-5-fluorocytidine by carboxylesterase in the liver and subsequently to 5′-deoxy-5-fluorouridine by cytidine deaminase (CDA) in liver and tumor cells. Then, it is metabolized to its active form (5-FU) by thymidine phosphorylase (TP), which is found in higher concentrations in tumor tissues than normal tissues [6]. In tumor and normal cells, 5-FU is then further metabolized into two active metabolites, which are 5-fluorouridine triphosphate (FUTP) and 5-fluoro-2′-deoxyuridine 5′-monophosphate (FdUMP), which influence their targets (DNA and RNA). Capecitabine and its metabolites are almost completely (~96%) excreted in urine and the rest through feces [6,7,8].

In recent years, much research has been conducted to identify different diagnostic and screening methods that could help in identifying genetic inter-individual variability markers, reducing the mortality of CRC, and improving the overall survival rate. This research has mostly focused on interfering with the genetic factors using well-known predicted CRC models to identify the genetic risk score (GRS) and polygenic risk score (PRS). The increasing number of genome-wide association studies (GWASs) on CRC has led to a progressive improvement regarding the impact of common genetic variants or single-nucleotide polymorphisms (SNPs) on the risk of CRC [9,10]. Many genetic variations exist among the enzymes involved in capecitabine metabolism, particularly in the mitochondrial thymidylate biosynthesis pathway. SNPs are the most popular genetic variations generally used as genetic biomarkers for predicting and identifying CRC development, interacting with treatment response, raising the risk of adverse drug reactions (ADR), and susceptibility recurrence of disease for patients [9,10]. Therefore, the frequencies of genotype and allele distributions for five SNPs in three major genes involved in capecitabine metabolism [Lys27Gln and Ala70Thr in cytidine deaminase (*CDA*), S471L in thymidine phosphorylase (*TP*), and C677T and A1298C in 5,10-methylenetetrahydrofolate reductase (*MTHFR*)] were examined in CRC patients and compared with control subjects to elucidate their contributions to CRC development risk. A polymerase chain reaction–restriction fragment length polymorphism (PCR-RFLP) assay was used for the analysis. This is a novel study performed on CRC patients treated with capecitabine in Jeddah, Saudi Arabia.

## 2. Materials and Methods

### 2.1. Materials

A QIAamp DNA Blood Mini Kit (250) and a DNA ladder (50 bp or 100 bp gene ruler) were purchased from Qiagen, Hilden, Germany. The HotStart-IT^®^ FideliTaq^TM^ PCR Master Mix (2X) was purchased from Affymetrix Inc., Santa Clara, CA, USA. FastDigest restriction enzymes were all purchased from Thermo Fisher Scientific Inc., Waltham, MA, USA. The PCR primers were obtained from Eurofins Genomics, Ebersberg, Germany.

### 2.2. Sample Collection

This study was approved by the Biomedical Ethical Committee of the Faculty of Medicine, King Abdulaziz University, Jeddah, Saudi Arabia, under reference number 261-15. All participants agreed to participate in this study by signing a consent form that followed Helsinki’s ethical agreement for research on human specimens. Whole blood samples (2 mL) were collected from each subject in lavender-top vacutainers. The total number of subjects (n) in this study was 131, with ages ranging from 33 to 85 years. They were divided into two groups: 66 CRC patients and 65 control subjects. Blood samples of CRC patients from the Day Care Unit of King Abdulaziz University Hospital and control subjects from the Blood Bank at King Fahad General Hospital in Jeddah were collected from April to November 2022. The following inclusion and exclusion criteria for participants in this case–control study were chosen: CRC patients were included if they had a biopsy- or colonoscopy-confirmed neoplasm with a well-documented demographic data including age, weight, height, hip and waist circumferences, family history of having CRC or any other tumor, drug and clinical history, and tumor pathology data, moreover, if they were able to provide a blood sample for genetic study (PCR-RFLP). All CRC patients (n = 66) were clinically classified according to the TNM classification system of malignant CRC tumors into either stages I and II (n = 17) or stages III and IV (n = 49). Regarding the treatment regimen, stage I patients did not receive any chemotherapy and had only surgery to remove the tumor, whereas patients in stages II and III had surgery and received adjuvant chemotherapy (2–3 weeks of either XELIRI or 3 weeks of XELOX). Stage IV patients with metastatic liver tumors received intensive adjuvant chemotherapy treatment for 3 weeks of XELIRI, XELOX, XELODA, and bevacizumab alongside the surgery. Any CRC patients who were non-Saudis and had CRC as a metastatic (non-primary) tumor were excluded from the study. On the other hand, for the controls, any individuals with a good overall health status, based on their clinical examination and laboratory investigations, and who had no family history of cancer or genetic disorders were included. However, those who were non-Saudis and had metabolic syndromes, chronic or genetic diseases were excluded from the study.

### 2.3. Genomic DNA Extraction and Quality Control

The QIAamp DNA mini kit (250) (Qiagen, Hilden, Germany) was used to extract genomic DNA (gDNA) from whole blood samples (200 μL), following the manufacturer’s instructions. By calculating the absorbance ratio (260/280 nm), the concentration and purity of the DNA were determined using a DS-11 Nanodrop spectrophotometer (DeNovix Inc, Wilmington, DE, USA). All extracted and purified DNA samples were stored at −20 °C for further analysis. The extraction and purification of gDNA samples were performed at the Cancer and Mutagenesis Research Unit, King Fahd Medical Research Center, King Abdulaziz University, Jeddah, Saudi Arabia.

### 2.4. Polymerase Chain Reaction

To prepare a 25 µL PCR reaction, 12.5 µL of HotStart-IT^®^ FideliTaq™ PCR Master Mix (2X), 1 µL of each (100 nmol) primer (forward and reverse), 8.5 µL of nuclease-free water, and 2 µL of (100 ng/μL) DNA template were added into PCR tubes. Then, the PCR tubes were run on an Applied Biosystems thermal cycler (Thermo Fisher Scientific Inc., Waltham, MA, USA) after the specific conditions were programmed for each specific variant (Table 1). The PCR primers were previously published [11,12,13]. The primers were as follows: for *CDA* Lys27Gln variant, forward primer (5′-GCGGTCCCAAAAGGGTCAGTTTGCTCCCAGGAGGCGAAG-3′) and reverse primer (5′-GCGGTCCCAAAAGGGTCAGTAGATTCTCCCCTCCTGGGT-3′); for *CDA* Ala70Thr variant, forward primer (5′-TGTCCTTCTCCCCACCTTG-3′) and reverse primer (5′-GGAAGATGTTGGCTAAAGAGATG-3′); for *TP* S471L variant, forward primer (5′-GCAGGAGGCGCTCGTACTCT-3′) and reverse primer (5′-GCCCAAGCACTGACAAGGTTT-3′); for *MTHFR* C677T variant, forward primer (5′-TGAAGGAGAAGGTGTCTGCGGGA-3′) and reverse primer (5′-AGGACGGTGCGGTGAGAGTG-3′); and finally for *MTHFR* A1298C variant, forward primer (5′-CTTTGGGGAGCTGAAGGACTACTAC-3′) and reverse primer (5′-CACTTTGTGACCATTCCGGTTTG-3′).

### 2.5. Polymorphism Analysis Using the RFLP Assay

Thermo Scientific FastDigest™ restriction enzymes (Thermo Fisher Scientific Inc., Waltham, MA, USA) were used for rapid PCR digestion, following the manufacturer’s instructions. RFLP was performed by adding 17 μL of nuclease-free water, 2 μL of 10X FastDigest Green Buffer, 10 μL of PCR product, and 1 μL of FastDigest enzymes into a 1.5 mL Eppendorf tube. The mixture was then gently stirred and incubated for 20 min in a water bath at 37 °C. A 5 μL Qiagen DNA ladder (50 or 100 gene ruler) and digest mixture were loaded into wells of either 12.5% polyacrylamide gel (for the A1298C variant in the *MTHFR* gene) or 2% agarose gel (for the other 4 SNPs) and were run on the electrophoresis apparatus for 30 min at 100–150 volts. To obtain an electronic image of the restricted bands, the gels were placed in a UV imaging apparatus. Table 1 lists the RFLP sizes of enzyme-restricted PCR products that have been described in past studies [11,12,13]. To confirm the results of RFLP, five randomly selected PCR-digested samples from each tested variant were sent for DNA sequencing at the Center of Excellence in Genomic Medicine Research at King Fahd Medical Research Center, King Abdulaziz University, Jeddah, Saudi Arabia.

### 2.6. Statistical Analysis

Using GraphPad Prism version 5.0, the Chi-square (χ^2^) test and two-tailed Fisher’s exact probability test were conducted to estimate the Hardy–Weinberg equilibrium (HWE) for the distribution of genotypic and allelic frequencies. The odds ratio (OR), risk ratio (RR), and 95% confidence interval (CI) were calculated using a 2 × 2 contingency table to estimate the strength of the association between genotype frequencies and CRC development risk. The comparison of physical characteristics between two groups was conducted by unpaired *t*-test with Welch’s correction. The statistical significance level was *p* < 0.05.

## 3. Results

### 3.1. The Physical Characteristics of the Study Groups

The physical characteristics of the 66 CRC patients and 65 control subjects were calculated using the unpaired *t*-test. Based on the gender of patients and controls, they were categorized into two groups. In the patient group, men were n = 50, which equals 76%, and women were n = 16, which equals 24%, whereas in the control group, the men were n = 43, representing 66%, and women were n = 22, representing 34%. The unpaired *t* test results between patients and controls regarding their physical characteristics showed a very high significant difference in weight and body mass index (BMI) (*p*-values were 0.0009 and 0.0002, respectively). Moreover, non-significant results (*p* ˃ 0.05) were observed between patients and controls in age, height, waist circumference, hip circumference, and waist-to-hip ratio (WHR).

### 3.2. The Lys27Gln Variant in the CDA Gene 

An example of the genotyping of the Lys27Gln variant in the *CDA* gene is shown in Supplementary Figure S1. Table 2 summarizes the Chi-square (χ^2^) test results for the genotype and allele frequencies of the *CDA* Lys27Gln for CRC patients and control subjects. In the CRC group, the genotypic frequencies were 42.4% (n = 28) wild type (AA), 39.4% (n = 26) heterozygous (AC), and 18.2% (n = 12) homozygous (CC). The frequency of the A allele was 62%, whereas the frequency of the C allele was 38%. Therefore, the genotype distribution was within the assumed HWE (χ^2^ = 1.97, degree of freedom [DF] = 1, *p* > 0.05). In the control group, the genotypic frequencies were 51% (n = 33) wild type (AA), 25% (n = 16) heterozygous (AC), and 24% (n = 16) homozygous (CC). The frequency of the A allele was 64%, whereas the frequency of the C allele was 36%. Therefore, the genotype distribution was outside the assumed HWE (χ^2^ = 14.72, DF = 1, *p* < 0.01). These results showed that heterozygous genotype (AC) carriers demonstrated a slight increase in OR (1.92 [95% CI = 0.86–4.27]) compared to wild-type (AA) carriers. 

### 3.3. The Ala70Thr Variant in the CDA Gene

An example of the genotyping of the Ala70Thr variant in the *CDA* gene is shown in Supplementary Figure S2. Table 2 summarizes the Chi-square (χ^2^) results for the genotype and allele frequencies of the *CDA* Ala70Thr for CRC patients and control subjects. In the CRC group, the genotypic frequencies were 32% (n = 21) wild type (GG), 59% (n = 39) heterozygous (GA), and 9% (n = 6) homozygous (AA). The frequency of the G allele was 62%, whereas the frequency of the A allele was 38%. Therefore, the genotype distribution was outside the HWE (χ^2^ = 3.85, DF = 1, 0.025 > *p* > 0.05). In the control group, the genotypic frequencies were 42% (n = 27) wild type (GG), 54% (n = 35) heterozygous (GA), and 4% (n = 3) homozygous (AA). The frequency of the G allele was 69%, whereas the frequency of the A allele was 31%. Therefore, the genotype distribution was outside the HWE assumption (χ^2^ = 4.07, DF = 1, 0.025 > *p* > 0.05). This variant presented a significant risk for CRC development based on the OR and RR values, as the homozygous genotype (AA) carriers showed an approximately three-fold higher risk compared to the wild-type (GG) carriers (OR = 2.57 [95% CI = 0.57–11.51] and RR = 2.22 [95% CI = 0.62–8.03]).

### 3.4. The S471L Variant in the TP Gene 

An example of the genotyping of the S471L variant in the *TP* gene is shown in Supplementary Figure S3. Table 3 summarizes the Chi-square (χ^2^) test results for the genotype and allele frequencies of *TP* S471L for CRC patients and control subjects. In the CRC group, the genotypic frequencies were 3% (n = 2) wild type (CC), 95.5% (n = 63) heterozygous (CT), and 1.5% (n = 1) homozygous (TT). The frequency of the C allele was 51%, whereas the frequency of the T allele was 49%. Therefore, the genotype distribution was outside the assumed HWE (χ^2^ = 54.6, DF = 1, *p* ˂ 0.01). The control group had no heterozygous (CT) or homozygous (TT) genotypes, since the genotypic frequencies were 100% (n = 65) wild type (CC). The frequency of the C allele was 100%. The genotype distribution was within the HWE (χ^2^ = 0, DF = 1, *p* > 0.05). Because all the control subjects had a normal genotype distribution (no recorded wild/mutant or mutant/mutant genotypes), the results revealed that the S471L variant had no significant effect on CRC development in our study population.

### 3.5. The C677T Variant in the MTHFR Gene

Table 4 summarizes the Chi-square (χ^2^) test results for the genotype and allele frequencies of *MTHFR* C677T in CRC patients and control subjects. In the CRC group, the genotypic frequencies were 82% (n = 54) wild type (CC), 9% (n = 6) heterozygous (CT), and 9% (n = 6) homozygous (TT). The frequency of the C allele was 87%, whereas the frequency of the T allele was 13%. Therefore, the genotype distribution was outside the assumed HWE (χ^2^ = 22.43, DF = 1, *p* < 0.01). In the control group, the genotypic frequencies were 94% (n = 61) wild type (CC), 6% (n = 4) heterozygous (CT), and 0% (n = 0) homozygous (TT). The frequency of the C allele was 97%, whereas the frequency of the T allele was 3%. The genotypes distribution was within the HWE assumption (χ^2^ = 0.06, DF = 1, *p* > 0.05). The results reported a significant difference in the carriers of homozygous genotype (TT) (*p* = 0.01), although the OR and RR values were not calculated due to the absence of homozygous genotype (TT) carriers in the control group. Furthermore, the results of the 2 × 2 contingency table demonstrated that patients with the recessive allele (T) had a higher risk of developing CRC than dominant-allele carriers (C).

### 3.6. The A1298C Variant in the MTHFR Gene

Table 4 summarizes the Chi-square (χ^2^) test results for the genotype and allele frequencies of *MTHFR* A1298C in CRC patients and control subjects. In the CRC group, the genotypic frequencies were 64% (n = 42) wild type (AA), 5% (n = 3) heterozygous (AC), and 31% (n = 21) homozygous (CC). The frequency of the A allele was 67%, whereas the frequency of the C allele was 33%. Therefore, the genotype distribution was outside the HWE (χ^2^ = 54.44, DF = 1, *p* < 0.01). In the control group, the genotypic frequencies were 59% (n = 38) wild type (AA), 29% (n = 19) heterozygous (AC), and 12% (n = 8) homozygous (CC). The frequency of the A allele was 73%, whereas the frequency of the C allele was 27%. The genotype distribution was outside the HWE assumption (χ^2^ = 4.58, DF = 1, 0.025 < *p* < 0.05). The findings indicated that the heterozygous genotype (AC) in this variant demonstrated a highly significant difference (*p* = 0.001) in terms of protecting participants from CRC development. However, the OR and RR values indicated a significant risk for CRC development, as the carriers of the homozygous genotype (CC) showed an approximately two-fold higher risk for CRC development compared to the wild-type carriers (AA) (OR = 2.38 [95% CI = 0.94–5.99] and RR = 1.92 [95% CI = 0.93–3.94]).

## 4. Discussion 

Worldwide, CRC is one of the most diagnosed cancer types (it ranked third in both genders, after breast and lung cancer, and representing 10% of cancer incidence [14]). In 2030, according to CRC’s global burden expectations, it is expected that CRC cases will be raised to 60% (more than 2 million new cases), while CRC mortality will be 1.1 million due to the increasing economic growth rate [1]. CRC has many risk factors that have been classified based on their sources. The first class of risk factors includes environmental factors, and the second class includes genetic factors, which represent 35% of CRC cases [10]. Efficient diagnostic methods are important to manage CRC patients’ responses, to assist them in the treatment plan with accurate means especially for people aged ≥ 50 years, and to increase the survival rate [15]. In the treatment of CRC, chemotherapy remains one of the most used treatment regimens in early and advanced CRC cases. In general, chemotherapy treatment is given to decrease or eliminate cancer cells, inhibit tumor growth and metastasis, and relieve pain. However, these drugs may cause relapse aggressiveness and may result in drug resistance due to the presence of cancer stem cells [16]. One of the drugs used in early and metastatic CRC is capecitabine. Each active metabolite of capecitabine, 5-FU, FUTP, and FdUMP, has a mechanism for affecting its targets and thus causing cell injury. One of the mechanisms is that during RNA synthesis, FUTP can mistakenly combine with nuclear transcriptional enzymes instead of uridine triphosphate (UTP), which results in affecting the RNA process and consequently affects the synthesis of protein. The other mechanism is blocking the pathway of nucleotide biosynthesis by inhibiting thymidylate formation from 2′-deoxyuridylate, which is an essential precursor of thymidine triphosphate (TTP), and thus cell division (tumor growth) is inhibited due to TTP deficiency that is necessary for DNA replication. This inhibition is achieved by covalent binding of FdUMP to thymidylate synthase with folate cofactor N5-10-methylenetetrahydrofolate to form a bound ternary complex and consequently blocking the formation of thymidylates [17]. The enzymes that are involved in the metabolism of capecitabine and its targets in the mitochondrial thymidylate biosynthesis pathway may contain many genetic variants that could affect their activity. SNPs are generally good genetic biomarkers for detecting CRC development, making some patients more susceptible to disease recurrence, or interfering with response to chemotherapy [18]. Moreover, it is well known that genetic variants in major oncogenes and tumor suppressor genes are responsible for cancer progression. However, these variants including SNPs can cause cancer in combination with other environmental factors [19]. Moreover, the effect of the SNPs may differ according to their location on the gene (coding or non-coding regions) and their type (synonymous or non-synonymous) [19,20]. For instance, coding SNPs can affect RNA processing, gene and protein modifications, or can interact with other SNPs to produce a stronger functional effect which could be pathogenic [19,20]. On the other hand, non-coding SNPs are more common than coding SNPs, and they can cause cancer through mechanisms including post-translational modifications of proteins and chromatin structure, regulation of genes transcription via proximal (cis) or distal (trans) interactions and weakening the binding capacity of transcription factors [19,20]. Regarding the five studied SNPs in the current study, less is known about their contribution to CRC progression as most of the published research was performed at the proteomic level (protein expression) and not on the genomic level (SNPs and variants). For instance, CDA activation alongside P53 over-expression was found to produce immune diversity and induce the class-switch of immunoglobulin genes in CRC adenoma patients. However, this expression was not correlated to patients’ 5-year survival, tumor stage, tumor size, and lymph node metastasis but was associated with tumor differentiation [21]. In silico analysis using publicly available human cancer projects showed that *CDA* Lys27Gln variant is correlated to CRC progression (COSMIC sample ID is TCGA-AM-5821-01). However, it was the only available human cancer project that explained the role of this SNP. The other four SNPs were not found in any of these human cancer projects on any of these platforms (COSMIC, TCGA, or Phosphositeplus). Moreover, a search on the Phosphositeplus platform showed that mutations in the *CDA*, *TP*, and *MTHFR* genes resulted in amino acid substitutions in the high-frequency level, which affect the function of these proteins (enzymes) in CRC cases (Supplementary file). Therefore, in this study, five major variants in three genes, namely *CDA* Lys27Gln, *CDA* Ala70Thr, *TP* S471L, *MTHFR* C677T, and *MTHFR* A1298C, were investigated and correlated to assess the effect of such variations on CRC development in patients treated with capecitabine (Xeloda^®^).

The first two variants investigated in this study were in the *CDA* gene. Cytidine deaminase (CDA) is one of the major enzymes in the metabolism of capecitabine in liver and tumor cells that deaminates cytidine and 2′-deoxycytidine for uracil derivative synthesis required for pyrimidine pathway preservation [6]. This enzyme is encoded by the *CDA* gene that is located on chromosome 1p36 [12]. Two variants (Lys27Gln and Ala70Thr) in the *CDA* gene have been shown to be associated with disease outcome in CRC capecitabine-treated patients [12]. In the current study, the Lys27Gln variant was found to have a slight risk effect with heterozygous genotype (AC) carriers, whereas Ala70Thr showed a significant risk of CRC development in homozygous genotype (AA) carriers. Most of the previous research on *CDA* variants has primarily focused on their correlation with ADR risk and drug-induced toxicities, especially those associated with capecitabine and gemcitabine [22]. A recent systematic review showed that only four variants of the dihydropyrimidine dehydrogenase (*DPYD*) gene are clinically relevant for the prediction of severe toxicity, and there are no validated predictive biomarkers of capecitabine effectiveness [23]. However, few studies have analyzed the correlation between these variants and the risk of developing cancers other than CRC. Zhou et al. have reported that Lys27Gln and Ala70Thr in the *CDA* gene had little association with lung cancer risk [12]. In contrast, a study on Japanese people by Sugiyama et al. found that *CDA* Lys27Gln had no correlation with cancer development [24]. Interestingly, no study has analyzed the correlation between Lys27Gln and Ala70Thr variants in the *CDA* gene and CRC development risk. Therefore, this study’s findings provide a novel contribution to the literature by analyzing the relationship between variants in genes that code important enzymes involved in capecitabine metabolism.

The third variant investigated in this study was S471L in the *TP* gene. Thymidine phosphorylase (TP) is one of the major enzymes in capecitabine’s metabolism that is found in tumor tissues at higher concentrations than normal tissues to produce active metabolite 5-FU, which has a role in preserving pyrimidine synthesis [6]. This enzyme is encoded by the *TP* gene that is located on chromosome 22q13 [11]. The results in the current study showed that all study subjects (controls) had a normal genotype distribution (CC) (no reported wild/mutant or mutant/mutant genotypes), which indicated that this variant had no significant effect on CRC development. Interestingly, a recent study by Jia et al. (2023) showed that the S471L variant in the *TP* gene might be used as a prognostic marker to predict the overall survival (OS) rate and disease-free survival (DFS) rate for CRC patients treated with capecitabine-based adjuvant chemotherapy through mediation of the mRNA expression of the *TP* gene [25]. Moreover, a study conducted by Jennings et al. (2013) found that the *TP* S471L (rs11479) variant had a significant association with early dose modifications and/or severe adverse events (adjusted OR = 2.02 [1.03–4.00], *p* = 0.042, adjusted OR = 2.70 [1.23–5.92], *p* = 0.01 respectively) [26]. 

The last two variants (C677T and A1298C) investigated in this study were in the *MTHFR* gene. The 5,10-methylenetetrahydrofolate reductase (*MTHFR)* gene on chromosome 1p36 encodes 5,10-methylenetetrahydrofolate reductase enzyme [13]. The MTHFR enzyme is responsible for the conversion of 5,10-methylenetetrahydrofolate (5,10-MTHF) to 5-methyltetrahydrofolate (5-MTHF), which is important for the folate–homocysteine cycle [27]. The 5,10-MTHF has a role in blocking the formation of thymidylates and synthesis of DNA by binding to thymidylate synthase with the FdUMP compound [6]. The two most popular *MTHFR* gene polymorphisms are C677T and A1298C, which are related to many diseases such as thrombosis, hypertension, and cancers [13]. The results of the current study showed that the C677T variant showed a significant impact on the carriers of homozygous genotype (TT); the OR and RR values were not calculated due to the absence of homozygous genotype (TT) carriers in the control group. Furthermore, the recessive allele (T) demonstrated a significantly high risk of CRC development. Conversely, for A1298C, the results showed that the heterozygous genotype (AC) of this variant demonstrated highly significant protection against CRC development, whereas homozygous genotype (CC) carriers experienced a high risk of CRC development. Past research has studied the correlation between this variant and CRC risk. In contrast to our results, Derwinger et al. conducted a retrospective analysis that reported that the C677T variant was not a risk factor for CRC [28]. Another meta-analysis study on the Asian population also found that this variant is not a risk factor for CRC [29]. Moreover, research on the Eastern Chinese Han population and worldwide population found that carriers of the homozygous genotype (TT) and the 677T allele in the C677T variant offered significant protection against CRC development [30,31,32]. However, one study reported that the risk of CRC development increased with decreasing folic acid levels in homozygous genotype (TT) carriers of the C677T variant [33]. A study by Ozen et al. on the Turkish population agreed with our findings and reported that the frequency of the T allele of the C677T variant was 4.2-fold higher in patients, and it correlated with an increased CRC risk [34]. A meta-analysis study indicated that the C677T variant showed a significant risk effect on CRC development in Asian, Caucasian, and mixed populations, whereas it had no significant risk effect on African populations [35]. Research conducted in the UK and France agrees with our finding that the A1298C variant is correlated with an increased risk of CRC development. Research conducted in the UK suggested that CRC development risk was lowered by increasing folic acid consumption [36,37]. Analysis of the allelic frequency distribution showed that the variant T allele of MTHFR C677T conferred a lower CRC susceptibility than did the wild-type C allele [38]. In contrast to our study, a meta-analysis study found that the A1298C variant was not significantly correlated with CRC development [31]. Research performed on the Asian population suggested that the C1298C genotype provided significant protection against CRC development [30,31,32]. In addition, Liu et al. (2019) performed a study to determine the impact of pharmacogenetics on predicting survival in gastroenteric cancer treated with capecitabine. They found that patients carrying homozygous genotype (CC) of *CDA* A79C (Lys27Gln) variant or homozygous genotype (CC) of *MTHFR* A1298C variants are not likely to benefit from capecitabine-based chemotherapy [39]. The findings revealed that the study population deviated from the HWE assumption. This could be because no alleles entered or left the population or underwent changes through mutation, and the migration or selection processes of mates were performed randomly [40]. The inconsistencies in the results regarding these variants effect on CRC risk could be due to ethnic disparities in populations, gender, age, lifestyle, and diet [31,34]. Dietary patterns and folic acid intake may have an influence on the enzymes responsible for methylation and DNA synthesis. Therefore, folic acid consumption strongly affects *MTHFR* polymorphisms [31,32,33]. Although the current study showed different impacts of the five SNPs on the risk of CRC development, it has some limitations. The most important limitation is the small size of samples. The second limitation is the lack of clinical investigation for enzymes activities and the molecular mechanisms by which those SNPs can increase CRC development risk in patients receiving capecitabine (pharmacogenomic analysis).

## 5. Conclusions

The current study’s findings reported that carriers of the heterozygous genotype (AC) of *CDA* Lys27Gln, carriers of the homozygous genotype (AA) of *CDA* Ala70Thr, and carriers of the homozygous genotype (CC) of *MTHFR* A1298C were associated with CRC risk. However, the analysis indicated no correlation between S471L in the *TP* gene and CRC development risk. Interestingly, the A1298C variant in the *MTHFR* gene with the heterozygous genotype (AC) provided highly significant protection against CRC development. Further examinations using tissues or serum samples are needed to elucidate the effect of such variants on the activity of the enzymes as well as their pharmacodynamics and pharmacokinetics. 

## Figures and Tables

**Table 1:** PCR thermal cycler conditions, PCR product sizes, and RFLP restriction enzymes and sizes.

Gene	SNP ID	PCR Conditions	PCR Product Sizes	Restriction Enzyme RFLP Sizes
*CDA*	Lys27Gln (rs2072671)	- Initial denaturation at 95 °C for 5 min. - 40 cycles of denaturation at 95 °C for 30 s, annealing at 58 °C for 45 s, and extension at 72 °C for 60 s, followed by a final extension at 72 °C for 6 min.	129 bp	- Thermo Fisher Scientific FastDigest (*MboII*) (catalog number: FD0824). - Wild type (AA) (81, 48 bp), heterozygous (AC) (129, 81, 48 bp), and homozygous (CC) (129 bp).
	Ala70Thr (rs60369023)	- Initial denaturation at 95 °C for 5 min. - 40 cycles of denaturation at 95 °C for 30 s, annealing at 60 °C for 45 s, and extension at 72 °C for 60 s, followed by a final extension at 72 °C for 6 min.	300 bp	- Thermo Fisher Scientific FastDigest (*CpoI*) (catalog number: FD0744). - Wild type (GG) (181, 119 bp), heterozygous (GA) (300, 181, 119 bp), and homozygous (AA) (300 bp).
*TP*	S471L (rs11479)	- Initial denaturation at 94 °C for 10 min. - 30 cycles of denaturation at 94 °C for 15 s, annealing at 59 °C for 20 s, and extension at 72 °C for 30 s, followed by a final extension at 72 °C for 7 min.	121 bp	- Thermo Fisher Scientific FastDigest (*MnlI*) (catalog number: FD1074). - Wild type (CC) (66, 55 bp), heterozygous (CT) (121, 66, 55 bp), and homozygous (TT) (121 bp).
*MTHFR*	C677T (rs1801133)	- Initial denaturation at 95 °C for 5 min. - 37 cycles of denaturation at 95 °C for 30 s, annealing at 61 °C for 30 s, and extension at 72 °C for 30 s, followed by a final extension at 72 °C for 10 min.	198 bp	- Thermo Fisher Scientific FastDigest (*HinfI*) (catalog number: FD0804). - Wild type (CC) (198 bp), heterozygous (CT) (198, 175, 23 bp), and homozygous (TT) (175 and 23 bp).
	A1298C (rs1801131)		163 bp	- Thermo Fisher Scientific FastDigest (*MboII*) (catalog number: FD0824). - Wild type (AA) (56, 31, 30, 28, 18 bp), heterozygous (AC) (163 bp), and homozygous (CC) (84, 31, 30, 18 bp).

CDA: cytidine deaminase; TP: thymidine phosphorylase; MTHFR: 5,10-methylenetetrahydrofolate reductase.

**Table 2:** Genotypes and alleles frequency of the *CDA* gene variants (Lys27Gln and Ala70Thr) in patient and control groups.

	Lys27Gln Variant					Ala70Thr Variant				
	Genotypes			Alleles		Genotypes			Alleles	
	Wild (AA)	Heterozygous (AC)	Homozygous (CC)	Dominant (A)	Recessive (C)	Wild (GG)	Heterozygous (GA)	Homozygous (AA)	Dominant (G)	Recessive (A)
Patient group frequency % (n = 66)	42.4% (n = 28)	39.4% (n = 26)	18.2% (n = 12)	62% (n = 41)	38% (n = 25)	32% (n = 21)	59% (n = 39)	9% (n = 6)	62% (n = 40)	38% (n = 26)
Control group frequency % (n = 65)	51% (n = 33)	25% (n = 16)	24% (n = 16)	64% (n = 31)	36% (n = 24)	42% (n = 27)	54% (n = 35)	4% (n = 3)	69% (n = 44)	31% (n = 21)
Fisher’s exact test *p*-value		0.16	0.82		1		0.36	0.28		0.47
Odds ratio (OR) (95% CI)	1 (Reference)	1.92 (0.86–4.27)	0.88 (0.36–2.18)	1 (Reference)	1.04 (0.51–2.11)	1 (Reference)	1.43 (0.69–2.97)	2.57 (0.57–11.51)	1 (Reference)	1.36 (0.66–2.79)
Risk ratio (RR) (95% CI)	1 (Reference)	1.47 (0.91–2.4)	0.92 (0.49–1.71)	1 (Reference)	1.03 (0.66–1.6)	1 (Reference)	1.15 (0.86–1.53)	2.22 (0.62–8.03)	1 (Reference)	1.22 (0.77–1.94)

**Table 3:** Genotype and allele frequencies of the *TP* gene variant (S471L) in patient and control groups.

	S471L Variant				
	Genotypes			Alleles	
	Wild (CC)	Heterozygous (CT)	Homozygous (TT)	Dominant (C)	Recessive (T)
Patient group frequency % (n = 66)	3% (n = 2)	95.5% (n = 63)	1.5% (n = 1)	51% (n = 34)	49% (n = 32)
Control group frequency % (n = 65)	100% (n = 65)	0% (n = 0)	0% (n = 0)	100% (n = 65)	0% (n = 0)
Fisher’s exact test *p*-value		˂0.0001	0.03		1
Odds ratio (OR) (95% CI)	1 (Reference)	Not applicable	Not applicable	1 (Reference)	Not applicable
Risk ratio (RR) (95% CI)	1 (Reference)	Not applicable	Not applicable	1 (Reference)	Not applicable

**Table 4:** Genotype and allele frequencies of the *MTHFR* gene variants (C677T & A1298C) in patient and control groups.

	C677T Variant					A1298C Variant				
	Genotypes			Alleles		Genotypes			Alleles	
	Wild (CC)	Heterozygous (CT)	Homozygous (TT)	Dominant (C)	Recessive (T)	Wild (AA)	Heterozygous (AC)	Homozygous (CC)	Dominant (A)	Recessive (C)
Patient group frequency % (n = 66)	82% (n = 54)	9% (n = 6)	9% (n = 6)	87% (n = 57)	13% (n = 9)	64% (n = 42)	5% (n = 3)	31% (n = 21)	67% (n = 43)	33% (n = 23)
Control group frequency % (n = 65)	94% (n = 61)	6% (n = 4)	0% (n = 0)	97% (n = 63)	3% (n = 2)	59% (n = 38)	29% (n = 19)	12% (n = 8)	73% (n = 47)	27% (n = 18)
Fisher’s exact test *p*-value		0.52	0.01		0.05		0.001	0.08		0.45
Odds ratio (OR) (95% CI)	1 (Reference)	1.69 (0.45–6.32)	Not applicable	1 (Reference)	4.97 (1.03–23.99)	1 (Reference)	0.14 (0.04–0.52)	2.38 (0.94–5.99)	1 (Reference)	1.4 (0.66–2.94)
Risk ratio (RR) (95% CI)	1 (Reference)	1.63 (0.48–5.48)	Not applicable	1 (Reference)	4.43 (1–19.73)	1 (Reference)	0.2 (0.06–0.63)	1.92 (0.93–3.94)	1 (Reference)	1.26 (0.75–2.1)

## Data Availability

All data that support the reported results can be found upon reasonable request.

## Supplementary Materials

The following supporting information can be downloaded at: https://www.mdpi.com/article/10.3390/curroncol30090583/s1, Figure S1: Lys27Gln variant genotyping in *CDA* gene; Figure S2: Ala70Thr variant genotyping in *CDA* gene; Figure S3: S471L variant genotyping in *TP* gene; Figure S4: The distribution of the major cytidine deaminase (CDA) mutations in colorectal cancer (CRC) human samples; Table S1: In-silico analysis of the major cytidine deaminase (CDA) mutations in colorectal cancer (CRC) human samples (cBioPortal for Cancer genomics); Figure S5: The distribution of the major thymidine phosphorylase (TP) mutations in colorectal cancer (CRC) human samples; Table S2: In-silico analysis of the major thymidine phosphorylase (TP) mutations in colorectal cancer (CRC) human samples (cBioPortal for Cancer genomics); Figure S6: The distribution of the major 5,10-methylenetetrahydrofolate reductase (MTHFR) mutations in colorectal cancer (CRC) human samples; Table S3: In-silico analysis of the major 5,10-methylenetetrahydrofolate reductase (MTHFR) mutations in colorectal cancer (CRC) human samples (cBioPortal for Cancer genomics); Figure S7: Protein mutation frequency in cytidine deaminase (CDA) in 4440 TCGA tumor samples from 15 cancer types; Figure S8: Protein mutation frequency in thymidine phosphorylase (TP) in 4440 TCGA tumor samples from 15 cancer types; and Figure S9: Protein mutation frequency in 5,10-methylenetetrahydrofolate reductase (MTHFR) in 4440 TCGA tumor samples from 15 cancer types.

## References

[1] Rawla P., Sunkara T., Barsouk A. (2019). Epidemiology of colorectal cancer: Incidence, mortality, survival, and risk factors. Prz. Gastroenterol..

[2] Globocan 2020. Colorectal Cancer. International Agency for Research on Caner; World Health Oraganization: The Global Cancer Observatory, Lyon, France. https://gco.iarc.fr/today/data/factsheets/cancers/10_8_9-Colorectum-fact-sheet.pdf.

[3] Globocan 2021. Saudi Arabia. International Agency for Research on Caner; World Health Oraganization: The Global Cancer Observatory, Lyon, France. https://gco.iarc.fr/today/data/factsheets/populations/682-saudi-arabia-fact-sheets.pdf.

[4] Zhu J., Kong W., Huang L., Bi S., Jiao X., Zhu S. (2023). Identification of immunotherapy and chemotherapy-related molecular subtypes in colon cancer by integrated multi-omics data analysis. Front. Immunol..

[5] Xie Y.H., Chen Y.X., Fang J.Y. (2020). Comprehensive review of targeted therapy for colorectal cancer. Signal Transduct. Target. Ther..

[6] DrugBank 2005. Capecitabine. https://www.drugbank.ca/drugs/DB01101.

[7] National Library of Medicine, NIH (2012). Capecitabine. LiverTox: Clinical and Research Information on Drug-Induced Liver Injury.

[8] Mizumoto Y., Yokoyama S., Matsuda K., Iwamoto H., Mitani Y., Tamura K., Nakamura Y., Murakami D., Oka M., Kobayashi Y. (2020). Modulation of capecitabine administration to improve continuity of adjuvant chemotherapy for patients with colorectal cancer: A phase II study. Mol. Clin. Oncol..

[9] Sassano M., Mariani M., Quaranta G., Pastorino R., Boccia S. (2022). Polygenic risk prediction models for colorectal cancer: A systematic review. BMC Cancer.

[10] McGeoch L., Saunders C.L., Griffin S.J., Emery J.D., Walter F.M., Thompson D.J., Antoniou A.C., Usher-Smith J.A. (2019). Risk prediction models for colorectal cancer incorporating common genetic variants: A systematic review. Cancer Epidemiol. Biomark. Prev..

[11] Kumagai Y., Sugiura Y., Sugeno H., Takebayashi Y., Takenoshita S., Yamamoto T. (2006). Thymidine phosphorylase gene mutation is not a primary cause of mitochondrial neurogastrointestinal encephalomyopathy (MNGIE). Intern. Med..

[12] Zhou M., Wan H.Y., Gao B.L., Ding Y.J., Jun R.X. (2012). Genetic polymorphisms of XPD and CDA and lung cancer risk. Oncol. Lett..

[13] Nefic H., Mackic-Djurovic M., Eminovic I. (2018). The Frequency of the 677C>T and 1298A>C polymorphisms in the methylenetetrahydrofolate reductase (MTHFR) gene in the population. Med. Arch..

[14] Sung H., Ferlay J., Siegel R.L., Laversanne M., Soerjomataram I., Jemal A., Bray F. (2021). Global cancer statistics 2020: GLOBOCAN estimates of incidence and mortality worldwide for 36 cancers in 185 countries. CA Cancer J. Clin..

[15] Siegel R.L., Miller K.D., Sauer A.G., Fedewa S.A., Butterly L.F., Anderson J.C., Cercek A., Smith R.A., Jemal A. (2020). Colorectal cancer statistics, 2020. CA Cancer J. Clin..

[16] Chen L., Yang F., Chen S., Tai J. (2022). Mechanisms on chemotherapy resistance of colorectal cancer stem cells and research progress of reverse transformation: A mini-review. Front. Med..

[17] FDA (2000). Xeloda (Capecitabine). https://www.accessdata.fda.gov/drugsatfda_docs/label/2001/20896S6lbl.pdf.

[18] Horvat M., Potočnik U., Repnik K., Kavalar R., Štabuc B. (2016). Single nucleotide polymorphisms as prognostic and predictive factors of adjuvant chemotherapy in colorectal cancer of stages I and II. Gastroenterol. Res. Pract..

[19] Yang W., Zhang T., Song X., Dong G., Xu L., Jiang F. (2022). SNP-target genes interaction perturbing the cancer risk in the post-GWAS. Cancers.

[20] Deng N., Zhou H., Fan H., Yuan Y. (2017). Single nucleotide polymorphisms and cancer susceptibility. Oncotarget.

[21] Li L., Su N., Cui M., Li H., Zhang Q., Yu N., Wu S., Cao Z. (2019). Activation-induced cytidine deaminase expression in colorectal cancer. Int. J. Clin. Exp. Pathol..

[22] Peters G.J., Giovannetti E., Honeywell R.J., Ciccolini J. (2019). Can cytidine deaminase be used as predictive biomarker for gemcitabine toxicity and response?. Br. J. Clin. Pharmacol..

[23] Cura Y., Pérez-Ramírez C., Sánchez-Martín A., Membrive-Jimenez C., Valverde-Merino M.I., González-Flores E., Morales A.J. (2023). Influence of single-nucleotide polymorphisms on clinical outcomes of capecitabine-based chemotherapy in colorectal cancer patients: A systematic review. Cancers.

[24] Sugiyama E., Lee S.-J., Lee S.S., Kim W.-Y., Kim S.-R., Tohkin M., Hasegawa R., Okuda H., Kawamoto M., Kamatani N. (2009). Ethnic differences of two non-synonymous single nucleotide polymorphisms in CDA gene. Drug Metab. Pharmacokinet..

[25] Jia X., Zhang T., Sun J., Lin H., Bai T., Qiao Y., Li Y., Li G., Li G., Peng X. (2023). Rs11479 in *thymidine phosphorylase* associated with prognosis of patients with colorectal cancer who received capecitabine-based adjuvant chemotherapy. Pharmacogenomics Pers. Med..

[26] Jennings B.A., Loke Y.K., Skinner J., Keane M., Chu G.S., Turner R., Epurescu D., Barrett A., Willis G. (2013). Evaluating predective pharmacogenetic signature of adverse events in colorectal cancer patients treated with fluoropyrimidines. PLoS ONE.

[27] Choi Y., Kim J.O., Shim S.H., Lee Y., Kim J.H., Jeon Y.J., Ko J.J., Lee W.S., Kim N.K. (2016). Genetic variation of methylenetetrahydrofolate reductase (MTHFR) and thymidylate synthase (TS) genes is associated with idiopathic recurrent implantation failure. PLoS ONE.

[28] Derwinger K., Wettergren Y., Odin E., Carlsson G., Gustavsson B. (2009). A study of the MTHFR gene polymorphism C677T in colorectal cancer. Clin. Color. Cancer.

[29] Rai V. (2015). Evaluation of the MTHFR C677T polymorphism as a risk factor for colorectal cancer in Asian populations. Asian Pac. J. Cancer Prev..

[30] Li H., Xu W., Shen H., Chen Q., Hui L., Long L., Zhu X. (2011). Methylenetetrahydrofolate reductase genotypes and haplotypes associated with susceptibility to colorectal cancer in an eastern Chinese Han population. Genet. Mol. Res..

[31] Zhou D., Mei Q., Luo H., Tang B., Yu P. (2012). The polymorphisms in methylenetetrahydrofolate reductase, methionine synthase, methionine synthase reductase, and the risk of colorectal cancer. Int. J. Biol. Sci..

[32] Zhao M., Li X., Xing C., Zhou B. (2013). Association of methylenetetrahydrofolate reductase C677T and A1298C polymorphisms with colorectal cancer risk: A meta analysis. Biomed. Rep..

[33] Ströhle A., Wolters M., Hahn A. (2005). Folic acid and colorectal cancer prevention: Molecular mechanisms and epidemiological evidence. Int. J. Oncol..

[34] Ozen F., Sen M., Ozdemir O. (2014). Methylenetetrahydrofolate reductase gene germ-line C677T and A1298C SNPs are associated with colorectal cancer risk in the Turkish population. Asian Pac. J. Cancer Prev..

[35] Teng Z., Wang L., Cai S., Yu P., Wang J., Gong J., Liu Y. (2013). The 677C>T (rs1801133) polymorphism in the MTHFR gene contributes to colorectal cancer risk: A meta-analysis based on 71 research studies. PLoS ONE.

[36] Küry S., Buecher B., Robiou-Du-Pont S., Scoul C., Colman H., Le Neel T., Le Houérou C., Faroux R., Ollivry J., Lafraise B. (2008). Low-penetrance alleles predisposing to sporadic colorectal cancers: A French case-controlled genetic association study. BMC Cancer.

[37] Lightfoot T.J., Barrett J.H., Bishop T., Northwood E.L., Smith G., Wilkie M.J., Steele R.J., Carey F.A., Key T.J., Wolf R. (2008). Methylene tetrahydrofolate reductase genotype modifies the chemopreventive effect of folate in colorectal adenoma, but not colorectal cancer. Cancer Epidemiol. Biomark. Prev..

[38] Lin K.-M., Yang M.-D., Tsai C.-W., Chang W.-S., Hsiao C.-L., Jeng L.-B., Yueh T.-C., Lee M.-C., Bau D.-T. (2018). The role of *MTHFR* genotype in colorectal cancer susceptibility in Taiwan. Anticancer. Res..

[39] Liu D., Li X., Li X., Zhang M., Zhang J., Hou D., Tong Z., Dong M. (2019). CDA and MTHFR polymorphisms are associated with clinical outcomes in gastroenteric cancer patients treated with capecitabine-based chemotherapy. Cancer Chemother. Pharmacol..

[40] Alghamdi J., Padmanabhan S. (2014). Fundamentals of complex trait genetics and association studies. Handbook of Pharmacogenomics and Stratified Medicine.

