# Supplementary material for: Genetic Variants in the Mitochondrial Thymidylate Biosynthesis Pathway Increase Colorectal Cancer Risk
Source: Curr Oncol. 2023 Aug 30;30(9):8039–53. doi: 10.3390/curroncol30090583 (PMC10529222; doi:10.3390/curroncol30090583)
Supplement: Supplementary file 1 [file curroncol-30-00583-s001.zip › curroncol-2432755-supplementary.pdf]

Supplementary file (Electrophoresis images and in-silico analysis)

Manuscript ID: curroncol-2432755

Manuscript title: Genetic variants in the mitochondrial thymidylate biosynthesis pathway increase colorectal cancer risk

Authors: Entesar M. Arrait, Ayat B. Al-Ghafari, Huda A. Al Doghaither

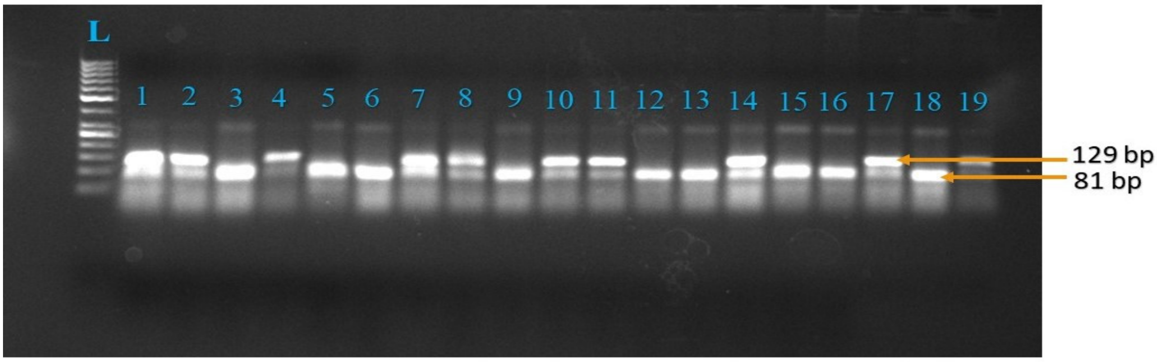

Figure S1. Lys27Gln variant genotyping in *CDA* gene. The PCR product size for this variant was 129 bp. These fragments were digested by *MboII* restricted enzyme, yielding the following: the amplicon of wild type (AA) genotype yielded two bands with sizes 81 and 48 bp (lanes 3, 5, 6, 9, 12, 13, 15, 16, and 18), the heterozygous (AC) genotype yielded three bands with sizes 129, 81 and 48 bp (lanes 1, 2, 4, 7, 8, 10, 11,14,17, and 19) and the homozygous (CC) genotype produced a band with a size 129 bp (lane 4). As shown from image, a size of 48 bp was difficult to be determined on 2% agarose gel while only two sizes 129 and 81 bp can be seen clearly.

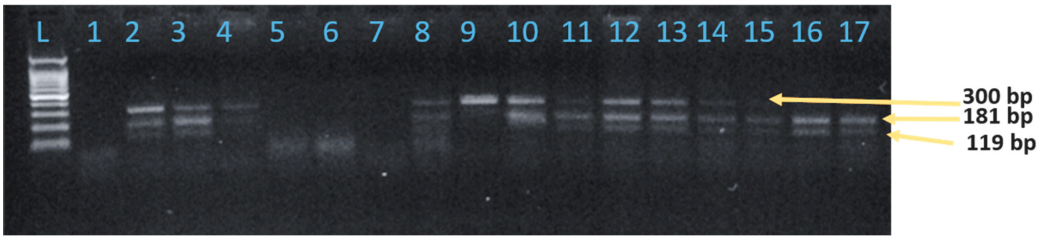

Figure S2. Ala70Thr variant genotyping in *CDA* gene. The PCR product size for the variant was 300 bp. These fragments were digested by *CpoI* restricted enzyme, yielding the following on 2% agarose gel: the amplicon of wild type (GG) genotype yielded two fragments with sizes 181 and 119 bp (lanes 16 and 17), the heterozygous (GA) genotype yielded three fragments with sizes 300, 181 and 119 bp (lanes 2, 3, 4, 8, 10, 11, 12, 13, 14, and 15) and the homozygous (AA) genotype produced a fragment with a size 300 bp (lane 9).

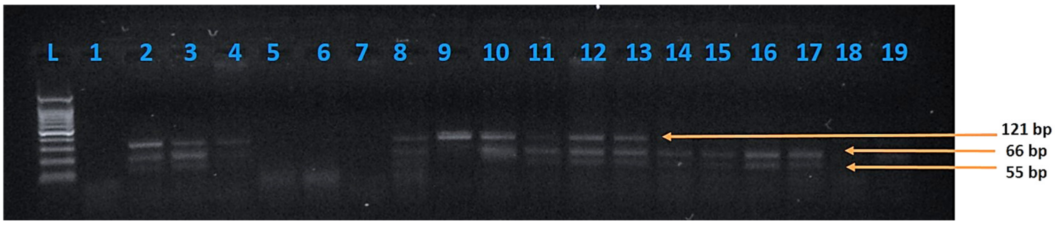

Figure S3. S471L variant genotyping in *TP* gene. The PCR product size for this variant was 121 bp. These fragments were digested by *MnlI* restricted enzyme, yielding the following on 2% agarose gel: the amplicon of wild type (CC) genotype yielded two fragments with sizes 66 and 55 bp (lanes 14-19), the heterozygous (CT) genotype yielded three fragments with sizes 121, 66 and 55 bp (lanes 2, 3, 8, 10, 11, 12, and 13) and the homozygous (TT) genotype produced a fragment with a size 121 bp (lane 9).

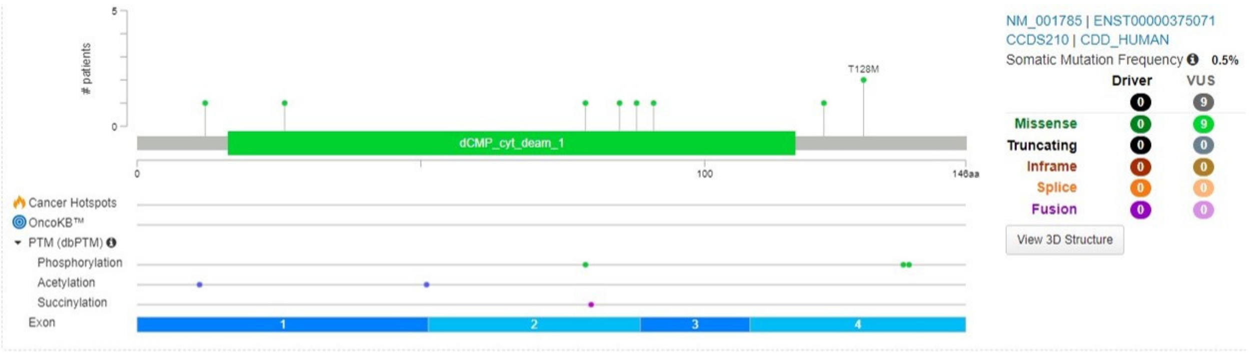

Figure S4. The distribution of the major cytidine deaminase (CDA) mutations in colorectal cancer (CRC) human samples. The image was obtained from cBioPortal for Cancer Genomics showing the major nine mutations in *CDA* gene among (n=1774 samples in 12 projects, and somatic mutation frequency 0.5%) and their locations on the gene exons. However, none of the nine missense mutations (M91V, T128M, A88T, K26N, I85T, D121Y, T128M, P12L, and Y79H) were considered as CRC hotspots.

Table S1. In-silico analysis of the major cytidine deaminase (CDA) mutations in colorectal cancer (CRC) human samples (cBioPortal for Cancer genomics).

Protein Change	Mutation Type	Variant Type	Chromosome	HGVSg	HGVSc	Exon	dbSNP
M91V	Missense	SNP	1	1:g.20940339A>G	ENST00000375071.3:c.271A>G	3	
T128M	Missense	SNP	1	1:g.20945003C>T	ENST00000375071.3:c.383C>T	4	rs139287750
A88T	Missense	SNP	1	1:g.20931528G>A	ENST00000375071.3:c.262G>A	2	rs150100090
K26N	Missense	SNP	1	1:g.20915700G>T	ENST00000375071.3:c.78G>T	1	rs17854868
I85T	Missense	SNP	1	1:g.20931520T>C	ENST00000375071.3:c.254T>C	2	rs2154532417
D121Y	Missense	SNP	1	1:g.20944981G>T	ENST00000375071.3:c.361G>T	4	
T128M	Missense	SNP	1	1:g.20945003C>T	ENST00000375071.3:c.383C>T	4	rs139287750
P12L	Missense	SNP	1	1:g.20915657C>T	ENST00000375071.3:c.35C>T	1	rs2101170695
Y79H	Missense	SNP	1	1:g.20931501T>C	ENST00000375071.3:c.235T>C	2	rs2154532408

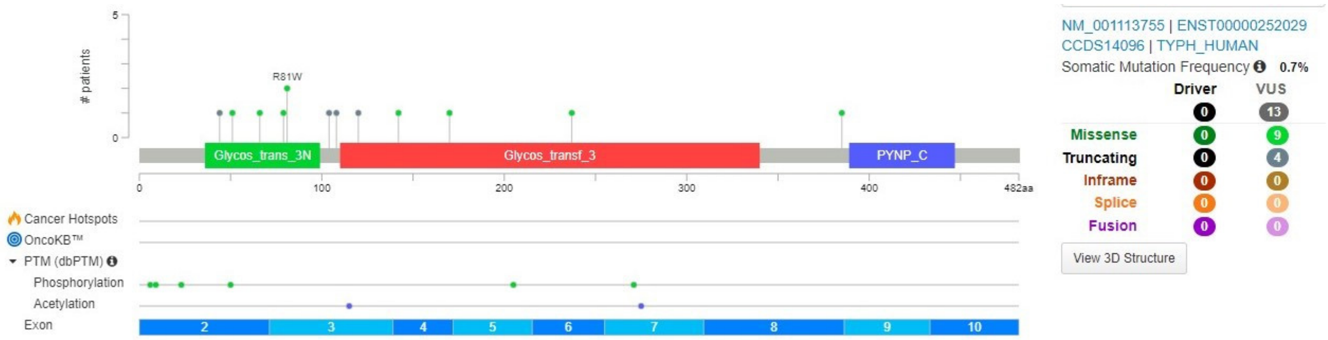

Figure S5. The distribution of the major thymidine phosphorylase (TP) mutations in colorectal cancer (CRC) human samples. The image was obtained from cBioPortal for Cancer Genomics showing the major 12 mutations in *TP* gene among (n=1774 samples in 12 projects, and somatic mutation frequency 0.7%) and their locations on the gene exons. Those mutations are classified as follows: [eight missense mutations (R81W, P170Q, A66V, G237R, M142I, E51K, R79Q, and A385T); three nonsense mutations (R44*, W108*, and W104*), and one frame shift deletion (G120Vfs*23). However, none of these mutations were considered as CRC hotspots.

Table S2. In-silico analysis of the major thymidine phosphorylase (TP) mutations in colorectal cancer (CRC) human samples (cBioPortal for Cancer genomics).

Protein Change	Mutation Type	Variant Type	Chromosome	HGVSg	HGVSc	Exon	dbSNP
R81W	Missense	SNP	22	22:g.50967741G>A	ENST00000252029.3:c.241C>T	3	
R81W	Missense	SNP	22	22:g.50967741G>A	ENST00000252029.3:c.241C>T	3	
R44*	Nonsense	SNP	22	22:g.50968009G>A	ENST00000252029.3:c.130C>T	2	
P170Q	Missense	SNP	22	22:g.50966948G>T	ENST00000252029.3:c.509C>A	4	
A66V	Missense	SNP	22	22:g.50967942G>A	ENST00000252029.3:c.197C>T	2	rs2069514092
G237R	Missense	SNP	22	22:g.50965650C>T	ENST00000252029.3:c.709G>A	6	
M142I	Missense	SNP	22	22:g.50967031C>T	ENST00000252029.3:c.426G>A	4	
E51K	Missense	SNP	22	22:g.50967988C>T	ENST00000252029.3:c.151G>A	2	rs768461642
G120Vfs*23	Frame_Shift_Deletion	DEL	22	22:g.50967623del	ENST00000252029.3:c.359del	3	
R79Q	Missense	SNP	22	22:g.50967746C>T	ENST00000252029.3:c.236G>A	3	rs751450304
W108*	Nonsense	SNP	22	22:g.50967658C>T	ENST00000252029.3:c.324G>A	3	
W104*	Nonsense	SNP	22	22:g.50967670C>T	ENST00000252029.3:c.312G>A	3	
A385T	Missense	SNP	22	22:g.50964681C>T	ENST00000252029.3:c.1153G>A	8	rs1253283946

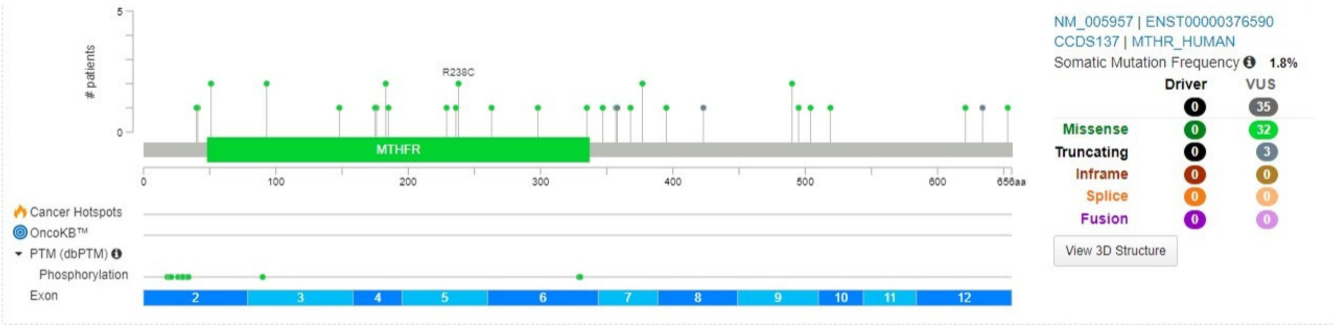

Figure S6. The distribution of the major 5,10-methylenetetrahydrofolate reductase (MTHFR) mutations in colorectal cancer (CRC) human samples. The image was obtained from cBioPortal for Cancer Genomics showing the major 35 mutations in *MTHFR* gene among (n=1774 samples in 12 projects, and somatic mutation frequency 1.8%) and their locations on the gene exons. Those mutations are classified as follows: [32 missense mutations (G490R, R183Q, R377H, R377C, F236L, R238C, R519C, P395S, R41Q, A175T, V93G, V93L, D495N, R335C, G298D, A368S, H263Q, G490R, V176A, L148R, R357H, R51Q, L229P, R183Q, E40K, R51W, R238C, G504R, L621P, L347I, E185D, and T653M); two nonsense mutations (R358* and Q634*), and one frame shift deletion (E423Rfs*3). However, none of these mutations were considered as CRC hotspots.

Table S3. In-silico analysis of the major 5,10-methylenetetrahydrofolate reductase (MTHFR) mutations in colorectal cancer (CRC) human samples (cBioPortal for Cancer genomics).

Protein Change	Mutation Type	Variant Type	Chromosome	HGVSg	HGVSc	Exon	dbSNP
G490R	Missense	SNP	1	1:g.11854026C>T	ENST00000376590.3:c.1468G>A	9	rs760349899
R183Q	Missense	SNP	1	1:g.11860307C>T	ENST00000376590.3:c.548G>A	4	rs574132670
R377H	Missense	SNP	1	1:g.11854822C>T	ENST00000376590.3:c.1130G>A	7	rs750323424
R377C	Missense	SNP	1	1:g.11854823G>A	ENST00000376590.3:c.1129C>T	7	rs121434296
F236L	Missense	SNP	1	1:g.11856335G>T	ENST00000376590.3:c.708C>A	5	rs34279942
E423Rfs*3	Frame_Shift_Deletion	DEL	1	1:g.11854495del	ENST00000376590.3:c.1267del	8	
R238C	Missense	SNP	1	1:g.11856331G>A	ENST00000376590.3:c.712C>T	5	rs377571071
R519C	Missense	SNP	1	1:g.11852412G>A	ENST00000376590.3:c.1555C>T	10	rs45496998
P395S	Missense	SNP	1	1:g.11854579G>A	ENST00000376590.3:c.1183C>T	8	rs1298093435
R41Q	Missense	SNP	1	1:g.11863052C>T	ENST00000376590.3:c.122G>A	2	rs775972969
A175T	Missense	SNP	1	1:g.11860332C>T	ENST00000376590.3:c.523G>A	4	rs1182635980
V93G	Missense	SNP	1	1:g.11861415A>C	ENST00000376590.3:c.278T>G	3	
V93L	Missense	SNP	1	1:g.11861416C>A	ENST00000376590.3:c.277G>T	3	
D495N	Missense	SNP	1	1:g.11854011C>T	ENST00000376590.3:c.1483G>A	9	rs749165790
R335C	Missense	SNP	1	1:g.11855183G>A	ENST00000376590.3:c.1003C>T	6	rs748289202
G298D	Missense	SNP	1	1:g.11855293C>T	ENST00000376590.3:c.893G>A	6	
A368S	Missense	SNP	1	1:g.11854850C>A	ENST00000376590.3:c.1102G>T	7	
H263Q	Missense	SNP	1	1:g.11855397G>T	ENST00000376590.3:c.789C>A	6	
G490R	Missense	SNP	1	1:g.11854026C>T	ENST00000376590.3:c.1468G>A	9	rs760349899
V176A	Missense	SNP	1	1:g.11860328A>G	ENST00000376590.3:c.527T>C	4	
L148R	Missense	SNP	1	1:g.11861250A>C	ENST00000376590.3:c.443T>G	3	
R357H	Missense	SNP	1	1:g.11854882C>T	ENST00000376590.3:c.1070G>A	7	rs977038830
R51Q	Missense	SNP	1	1:g.11863022C>T	ENST00000376590.3:c.152G>A	2	rs201618781
L229P	Missense	SNP	1	1:g.11856357A>G	ENST00000376590.3:c.686T>C	5	rs1362436529
R183Q	Missense	SNP	1	1:g.11860307C>T	ENST00000376590.3:c.548G>A	4	rs574132670
R358*	Nonsense	SNP	1	1:g.11854880G>A	ENST00000376590.3:c.1072C>T	7	rs377443637
E40K	Missense	SNP	1	1:g.11863056C>T	ENST00000376590.3:c.118G>A	2	
R51W	Missense	SNP	1	1:g.11863023G>A	ENST00000376590.3:c.151C>T	2	rs764131110
R238C	Missense	SNP	1	1:g.11856331G>A	ENST00000376590.3:c.712C>T	5	rs377571071
G504R	Missense	SNP	1	1:g.11853984C>T	ENST00000376590.3:c.1510G>A	9	rs1373785177
L621P	Missense	SNP	1	1:g.11850846A>G	ENST00000376590.3:c.1862T>C	12	
Q634*	Nonsense	SNP	1	1:g.11850808G>A	ENST00000376590.3:c.1900C>T	12	
L347I	Missense	SNP	1	1:g.11854913G>T	ENST00000376590.3:c.1039C>A	7	
E185D	Missense	SNP	1	1:g.11860300C>A	ENST00000376590.3:c.555G>T	4	
T653M	Missense	SNP	1	1:g.11850750G>A	ENST00000376590.3:c.1958C>T	12	rs35737219

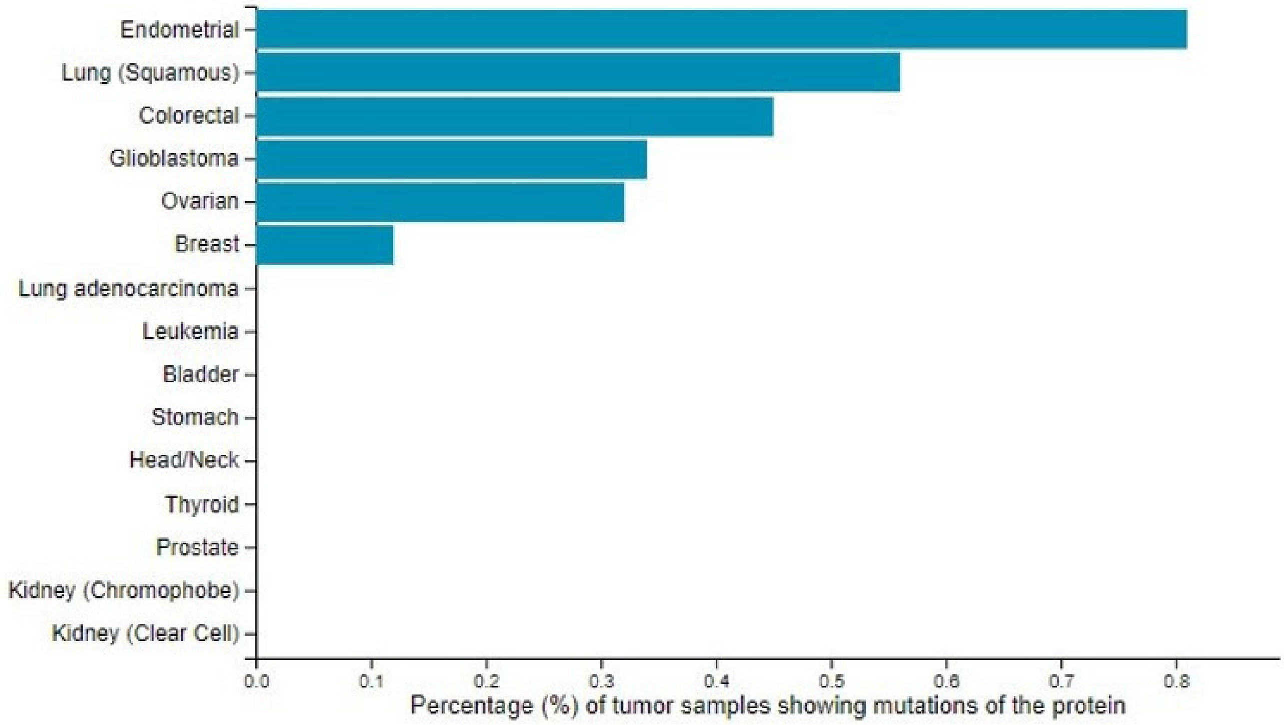

Figure S7. Protein mutation frequency in cytidine deaminase (CDA) in 4440 TCGA tumor samples from 15 cancer types. (source: https://www.phosphosite.org/proteinAction.action?id=1911500&showAllSites=true).

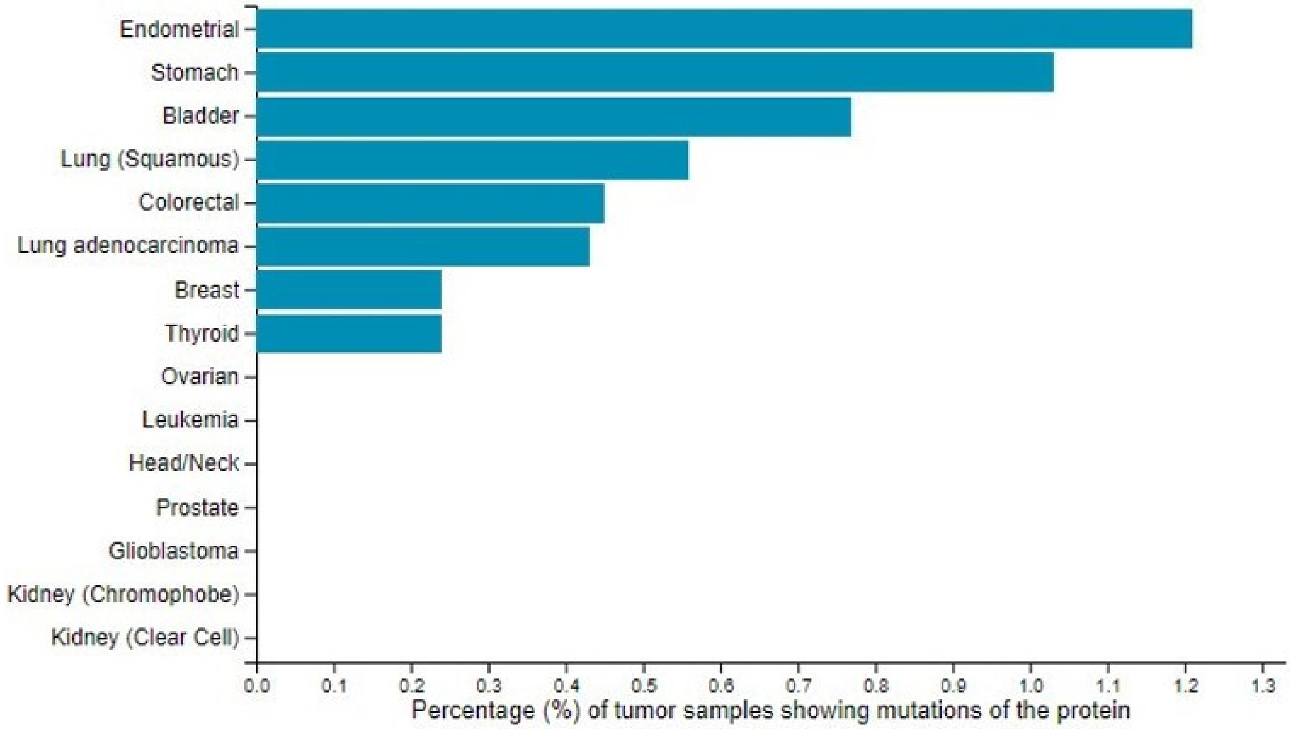

Figure S8. Protein mutation frequency in thymidine phosphorylase (TP) in 4440 TCGA tumor samples from 15 cancer types (source: https://www.phosphosite.org/proteinAction.action?id=1290138&showAllSites=true).

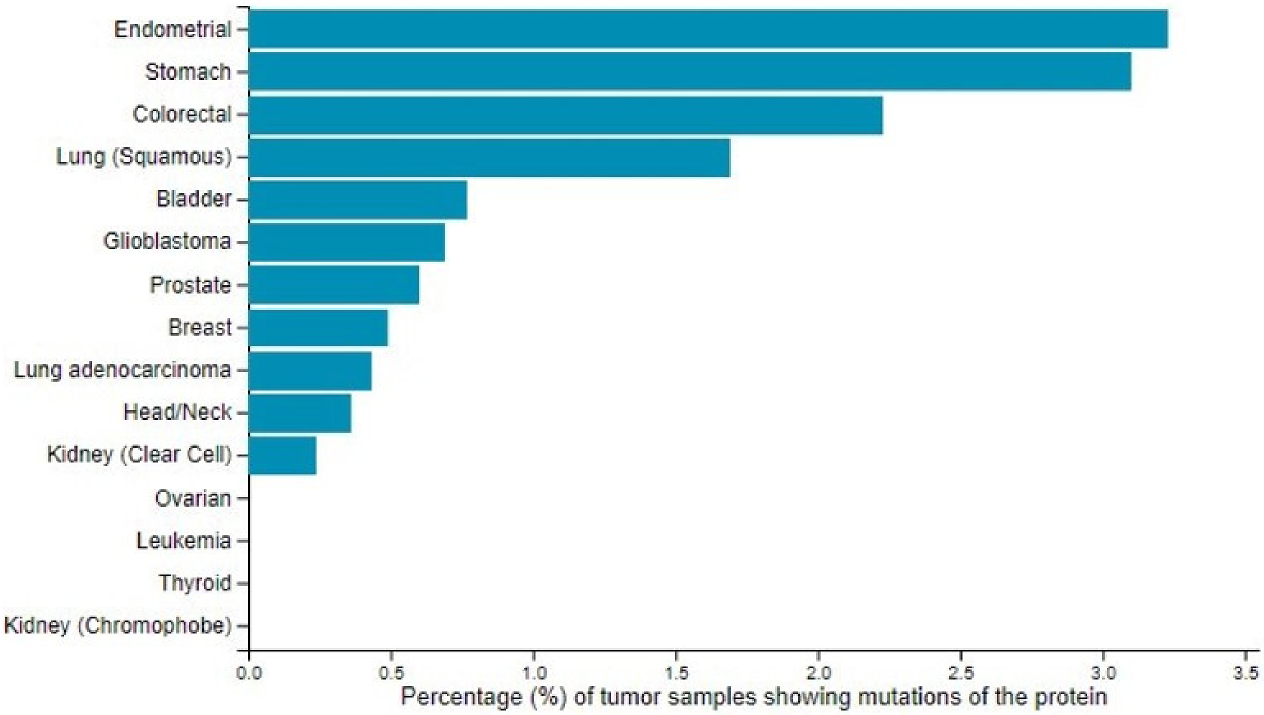

Figure S9. Protein mutation frequency in 5,10-methylenetetrahydrofolate reductase (MTHFR) in 4440 TCGA tumor samples from 15 cancer types (source: https://www.phosphosite.org/proteinAction.action?id=7717&showAllSites=true).
